# Improving Care Transitions in Alberta: Assessing the Effects of Introducing a Provincial Clinical Information System on Hospital-to-Home Transitions.

**DOI:** 10.23889/ijpds.v9i5.2585

**Published:** 2024-09-18

**Authors:** Tanmay Patil, Robin Walker, Congshi Shi, Wanning Song, Judy Seidel, Scott Oddie

**Affiliations:** 1 Alberta Health Services; 2 Red Deer Polytechnic

## Objectives

Transitioning from hospitals to primary care brings significant challenges, including increased readmission rates, mortality, and costs due to information gaps. Alberta, Canada, addressed this by integrating 1000+ health systems and implementing Connect Care (CC), a Clinical Information System (CIS), to enhance patient safety and care coordination. In 2020, the Primary Health Care Integration Network (PHCIN) released the Home to Hospital to Home (H2H2H) Transitions Guideline and metrics to improve patient outcomes and system integration.

## Approach

This study examines provincial data on H2H2H transitions measures from acute care hospitals using CC between April 1, 2022, and October 31, 2023, to assess transitions and support improvement efforts. CC data assesses transition components, including confirming primary care providers at hospital discharge, utilizing the LACE Readmission Risk Index, and ensuring timely discharge summary (DS) signoffs. It links with administrative data to evaluate post-discharge outcomes like primary care physician follow-up, unplanned readmissions, and Emergency Department (ED) visits.

## Results

47 CC-implemented sites show nearly 80% discharges identified a primary care provider; less than 5% incorporated the LACE index in DS. About 90-93% of DS were signed within 24-72 hours. Approximately 62% of moderate-risk and 55% of high-risk discharges received timely follow-up. Readmission and ED visit rates within 7-30 days varied from 2-8% and 5-11% respectively.

## Conclusions

In conclusion, adopting CC and H2H2H transition metrics facilitates integrated care measurement, highlighting the need for risk index inclusion and high-risk discharge follow-up for improved patient transitions. Sustained initiatives are vital for optimal outcomes and system integration in Alberta.

